# Panobinostat, a histone deacetylase inhibitor, suppresses leptomeningeal seeding in a medulloblastoma animal model

**DOI:** 10.18632/oncotarget.18132

**Published:** 2017-05-24

**Authors:** Ji Hoon Phi, Seung Ah Choi, Pil Ae Kwak, Ji Yeoun Lee, Kyu-Chang Wang, Do Won Hwang, Seung-Ki Kim

**Affiliations:** ^1^ Division of Pediatric Neurosurgery, Pediatric Clinical Neuroscience Center, Seoul National University Children's Hospital, Seoul, Korea; ^2^ Adolescent Cancer Center, Seoul National University Cancer Hospital, Seoul, Korea; ^3^ Department of Neurosurgery, Seoul National University Hospital, Seoul National University College of Medicine, Seoul, Korea; ^4^ Department of Anatomy, Seoul National University Hospital, Seoul, Korea; ^5^ Department of Nuclear Medicine, Seoul National University College of Medicine, Seoul, Korea

**Keywords:** medulloblastoma, leptomeningeal seeding, histone deacetylase inhibitor, anti-cancer therapy, bioluminescence imaging

## Abstract

Leptomeningeal seeding is a strong negative prognostic factor for medulloblastoma (MB). The mechanism of leptomeningeal seeding is unclear but may involve epigenetic regulation. In this study, we evaluated the feasibility of a histone deacetylase (HDAC) inhibitor, panobinostat, in the suppression of MB leptomeningeal seeding.

Panobinostat decreased the cell viability and proliferation, inducing cell cycle arrest and apoptosis in MB cell lines. The migration and adhesion capabilities were significantly decreased. Panobinostat effectively down-regulated protein expression of CCND1 and ID3 which has been associated with leptomeningeal seeding of MB. After panobinostat treatment, neurophil-like cellular processes developed and expression of synaptophysin and NeuroD1 was increased, indicating neuronal differentiation. In MB leptomeningeal seeding *in vivo* model, the panobinostat-treated group showed significantly decreased spinal leptomeningeal seeding and a survival benefit.

The findings demonstrate that panobinostat suppresses MB leptomeningeal seeding through the down-regulation of ID3 and the induction of neuronal differentiation. An HDAC inhibitor might be a potent treatment option for the treatment of MB patients with leptomeningeal seeding.

## INTRODUCTION

Medulloblastoma (MB) is the most common malignant brain tumor in children. The treatment outcomes of MB have been greatly improving for decades, but still a significant portion of patients suffer from tumor recurrence, which usually leads to fatality [1, 2]. One of the most distinct biological features of MB is its unique seeding pattern. MB predominantly spreads through the cerebrospinal fluid (CSF) into the leptomeningeal layers of the brain and spinal cord [3]. Approximately 30–40% of MB patients already have leptomeningeal seeding of tumors at diagnosis [2]. This leptomeningeal seeding is a strong negative prognostic factor of the disease [3]. Furthermore, leptomeningeal seeding is also frequently accompanied with recurrent MB, and it conveys a dismal prognosis because there are few therapeutic options for such patients [4].

The mechanism of MB leptomeningeal seeding is poorly understood. Although Group 3 MB, especially tumors with *c-myc* amplification, is associated with a higher rate of leptomeningeal seeding and worse prognosis, no driver mutation has been found for this phenotype [5]. Previously, we reported that the overexpression of inhibitor of differentiation (ID) 3 is involved in MB leptomeningeal seeding in human tumor tissues and animal models [6]. Interestingly, the ID3 mutation has not been reported in MB. Therefore, it is highly plausible that epigenetic mechanisms are responsible for the MB leptomeningeal seeding.

Histone deacetylase (HDAC) inhibitors are a promising group of anti-cancer drugs that change the gene expression patterns of cancer cells by an epigenetic modulation. The feasibility of HDAC inhibitors has been tested in a variety of human cancers, including malignant brain tumors. Panobinostat (LBH589) is a pan-HDAC inhibitor that shows a strong anti-proliferative effect in various cancer cell lines [7, 8]. It also regulates diverse cellular processes, including DNA repair and protein degradation [9]. For these pleiotropic anti-cancer effects, panobinostat has been approved for clinical use in multiple myeloma in the United States and European Union [9]. Malignant brain tumors can also be a target of panobinostat, which can penetrate the blood–brain barrier (BBB) [10]. A clinical trial on the combined use of panobinostat and bevacizumab on recurrent glioblastoma has been completed [11]. Panobinostat showed a substantial potency against diffuse intrinsic pontine glioma cells *in vitro* and *in vivo* [12]. Panobinostat also displayed a robust suppressive effect on an MB cell line [13]. However, the effect of HDAC inhibitors on MB leptomeningeal seeding has not been investigated in the appropriate models.

The effects of panobinostat include repression of stemness and the induction of differentiation [14]. It has been demonstrated that panobinostat triggers terminal myeloid differentiation of leukemic cells [15]. MB is an embryonic-type tumor composed of morphologically undifferentiated cells, but the expression of neuronal differentiation markers is commonly observed. Actually, differentiation-targeted therapies have been tried for MB with various agents, such as phenylacetate and retinoic acid [16, 17]. We postulated that HDAC inhibitors can suppress MB leptomeningeal seeding through the down-regulation of ID3 and the induction of neuronal differentiation.

## RESULTS

### Panobinostat inhibits MB cell growth, induces cell cycle arrest and apoptotic cell death

**Figure 1:**
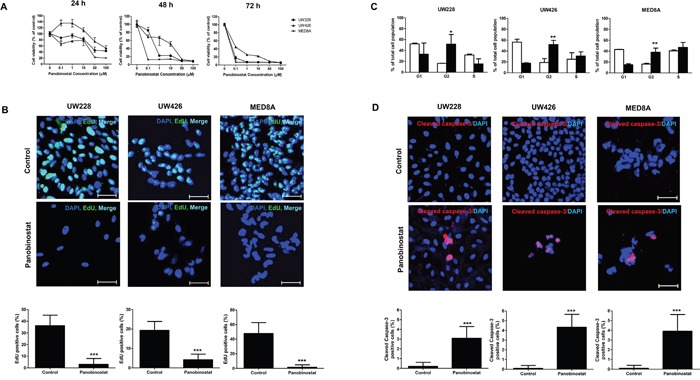
The anti-cancer effects of panobinostat on medulloblastoma (MB) cells **(A)** The number of viable MB cells decreases with increasing doses of panobinostat over time as measured by the CCK assay. **(B)** EdU incorporation assay shows that panobinostat decreases the EdU positive cells. Scale bars, 50 μm. Cells are counterstained with 4′,6′-diamidino-2-phenylindole (blue). **(C)** FACS analysis shows that treatment of MB cells with panobinostat induces G2/M cell cycle arrest. **(D)** Increased numbers of apoptotic cells are observed after panobinostat treatment. *p<0.05, **p<0.01, ***p<0.001.

To assess the anti-cancer effect of panobinostat on MB cells, we performed dose-response studies using a cell counting kit (CCK). We observed that panobinostat potently decreased cell viability in dose-dependent and time-dependent manners (Figure 1A). The IC_50_ values at 72 h were 0.054 ± 0.002 μM, 0.067 ± 0.016 μM, and 0.046 ± 0.002 μM in UW228, UW426 and MED8A, respectively (Table 1). The EdU assay showed that the proliferative cells were significantly reduced after panobinostat treatment at 48 h (control vs. panobinostat: 36.2 ± 8.99 % vs. 3.1 ± 4.98 % in UW228, p<0.001; 19.3 ± 4.52 % vs. 4.2 ± 2.90 % in UW426, p<0.001; 47.9 ± 14.87 % vs. 1.67 ± 3.24 % in MED8A, p<0.001; Figure 1B). To further explore the sensitivity of MB cells to panobinostat, we examined cell cycle changes invoked by panobinostat. Compared with the control, panobinostat caused the accumulation of cells with G2-M DNA content at 48 h (control vs. panobinostat: 16.28 ± 0.38 % vs. 51.59 ± 17.89 % in UW228, p=0.013; 18.89 ± 7.25 % vs. 51.77 ± 7.87 % in UW426, p=0.004; 16.31 ± 1.59 % vs. 38.05 ± 7.48 %, in MED8A, p=0.005; Figure 1C). The number of cells expressing cleaved caspase-3 increased after treatment of panobinostat in all MB cell lines tested, indicating that significant levels of apoptosis were induced by panobinostat (control vs. panobinostat: 0.2 ± 0.42 vs. 3.2 ± 1.20 in UW228, p<0.001; 0.1 ± 0.30 vs. 4.3 ± 1.3 in UW426, p<0.001; 0 ± 0 vs. 3.9 ± 1.73 in MED8A, p<0.001; Figure 1D).

**Table 1:** Comparison of the half maximal inhibitory concentration (IC_50_) for panobinostat in medulloblastoma cells

Hours	UW228	UW426	MED8A
24 h	15.167 ± 1.863	15.161 ± 0.252	10.686 ± 0.694
48 h	4.534 ± 0.234	10.457 ± 1.592	0.326 ± 0.282
72 h	0.054 ± 0.002	0.067 ± 0.016	0.046 ± 0.002

The IC_50_ value was calculated as the mean ± standard deviation (μM).

### Panobinostat affects the expression of CCND1, IDs and synaptophysin in MB cells

**Figure 2:**
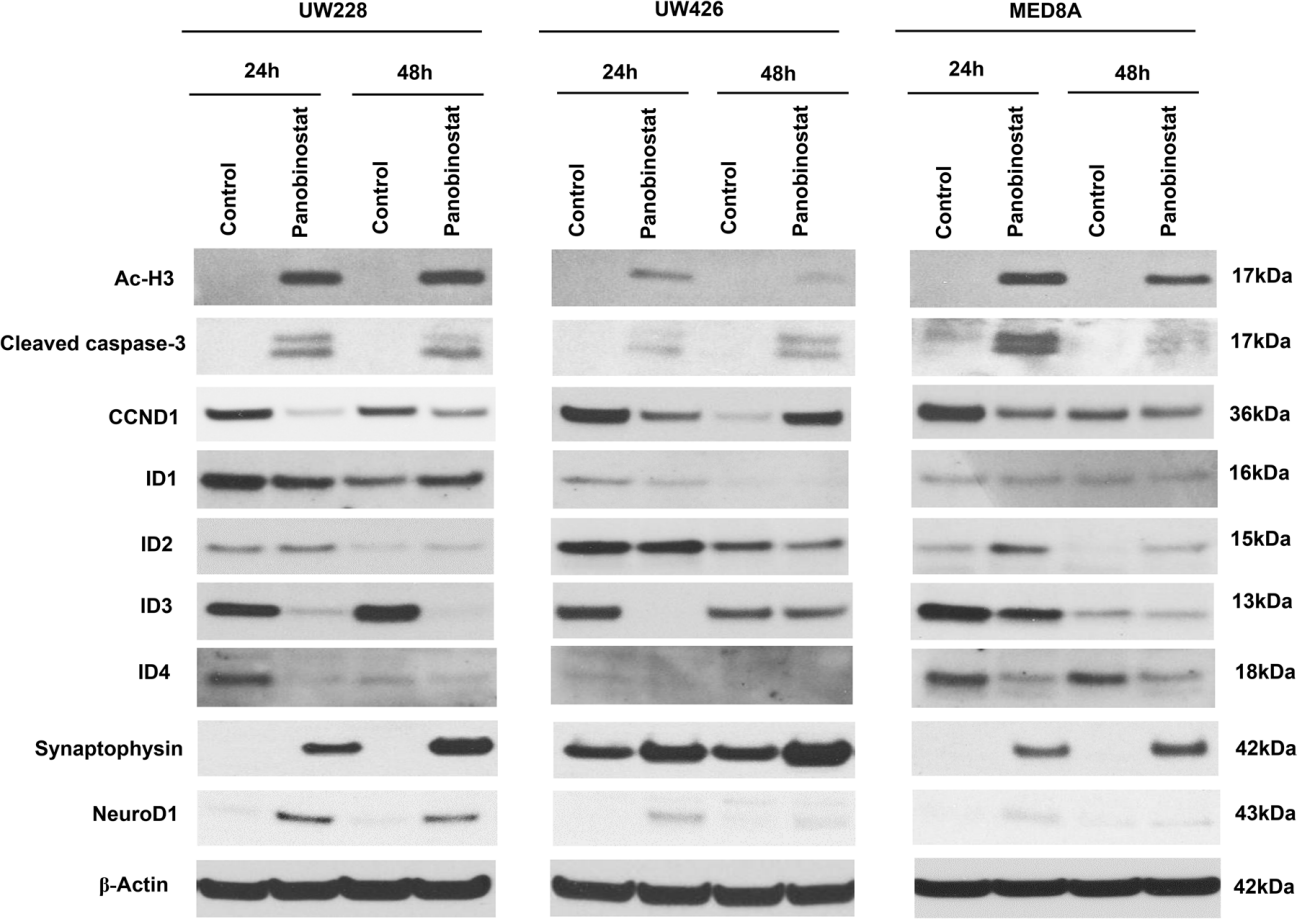
Effect of panobinostat on histone acetylation, apoptosis, cell cycle, IDs and differentiation-related signal pathway in medulloblastoma cells Immunoblotting of whole cell lysates prepared from MB cells treated with panobinostat. The representative western blot result shows panobinostat clearly regulated acetyl-Histone H3 (Ac-H3), cleaved caspase-3, CCND1, IDs, synaptophysin and NeuroD1.

To explore the mechanisms that influence MB cell proliferation and seeding, we evaluated the effect of panobinostat on protein expression levels [acetyl-Histone H3 (Ac-H3), cleaved capase-3, CCND1, IDs and differentiation related proteins, Figure 2]. Panobinostat effectively induced expression of Ac-H3 in all MB cell lines tested. Panobinostat treatment resulted in a significant level of apoptosis as observed by the increased level of cleaved caspase-3. One of the key cell-cycle regulators, CCND1, was significantly decreased by panobinostat. The observed G2 arrest in the cell cycle analysis could be explained by this decrease of CCND1.

Previously, we reported that ID3 was highly expressed in human MB tissues [6]. ID3 belongs to the ID protein family that regulates cell proliferation. Specifically, the suppression of ID3 in MB cells resulted in a significant increase of G2 arrest [6]. Therefore, we assessed the expression of the ID genes, ID1, ID2, ID3, and ID4, after panobinostat treatment. As expected, panobinostat robustly decreased the expression of ID3 and ID4 in all cell lines tested. The decreased expression of ID3 and ID4 in 24 hr was maintained at 48 hr. There were changes in ID1 and ID2 expression, but with less consistency in different cell lines. ID1 expression decreased in UW228 and UW426, but not in MED8A at 24 hr. The decreased ID1 expression in UW228 was even restored in 48 hr. The decrease of ID2 expression was observed only in UW426. The result supports more specific roles of ID3 in MB [6]. ID4 exhibited a concurrent regulation pattern with ID3, suggesting redundant roles for these proteins [6]. ID proteins are also involved in the maintenance of stemness characteristics. In neural stem cells, ID genes inhibited neuronal differentiation, keeping them in a stem cell state [18]. After panobinostat treatment, the expressions of synaptophysin and NeuroD1 were augmented in MB cells, which is consistent with the decreased ID3 expression.

### Panobinostat significantly suppresses cell migration and adhesion ability

**Figure 3:**
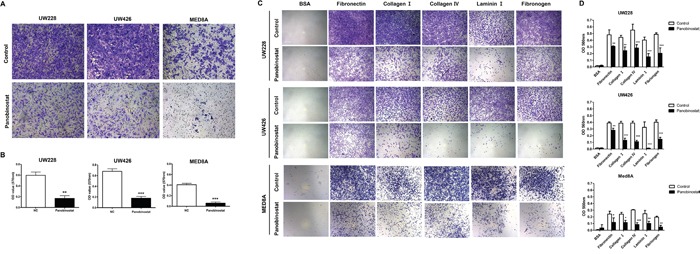
Effect of panobinostat on migration and adhesion ability of medulloblastoma cells Representative bright–field images of **(A)** migrated cells and **(B)** adhered cells are shown. Quantitative analysis reveals that panobinostat inhibits the **(C)** migration and **(D)** adhesion ability of MB cells. *p<0.05, **p<0.01, ***p<0.001.

A previous study showed that ID3 was highly related to MB leptomeningeal seeding *in vitro* and *in vivo* [6]. To investigate the effect of panobinostat on leptomeningeal seeding, we performed assays for cellular migration and adhesion, which are crucial capabilities of metastatic tumor cells. We first analyzed the migration ability of MB cells after panobinostat treatment. Panobinostat-treated MB cells exhibited significantly decreased migrated fractions compared with the controls (p=0.002 in UW228, p<0.001 in UW426 and p<0.001 in MED8A; Figure 3A and 3B). Second, the adhesion assays were used to observe the adhesive strength of MB cells binding to 5 major extracellular substrates: fibronection (p<0.01 in UW228, p<0.01 in UW426 and p<0.05 in MED8A), collagen I (p<0.001 in UW228, p<0.001 in UW426 and p<0.05 in MED8A), collagen IV (p<0.001 in UW228, p<0.001 in UW426 and p<0.001 in MED8A), laminin I (p<0.001 in UW228, p<0.001 in UW426 and p<0.01 in MED8A), and fibrinogen (p<0.001 in UW228, p<0.001 in UW426 and p<0.01 in MED8A). The adhesion capability of MB cells was remarkably decreased for all extracellular materials tested (Figure 3C and 3D).

### Panobinostat induces neuronal differentiation

**Figure 4:**
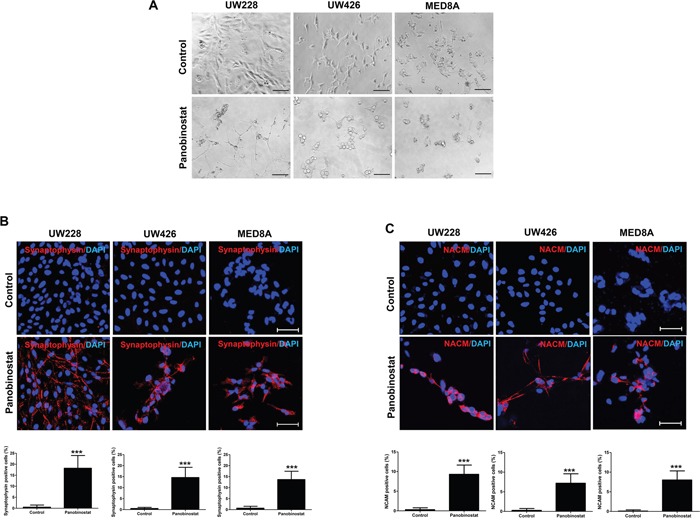
Triggered differentiation by panobinostat in medulloblastoma cells **(A)** Representative images show the typical morphology of neuronal differentiated cells. Panobinostat treated cells are positive for **(B)** synaptophysin (red) and **(C)** NCAM (red). Scale bars, 50 μm. Cells were counterstained with 4′,6′-diamidino-2-phenylindole (blue). ***p<0.001.

After treatment with panobinostat, morphological alterations were monitored. In panobinostat-treated UW-228 cells, extended cellular branches, reminiscent of axons and dendrites of differentiated neurons, were observed (Figure 4A). The morphological change was not conspicuous in other MB cell lines, but all of the cell lines expressed the neuronal makers synaptophysin (p<0.001 in all MB cells, Figure 4B) and neural cell adhesion molecule (NCAM, p<0.001 in all MB cells, Figure 4C) after panobinostat treatment.

### Panobinostat reduces MB leptomeningeal seeding and prolongs survival

**Figure 5:**
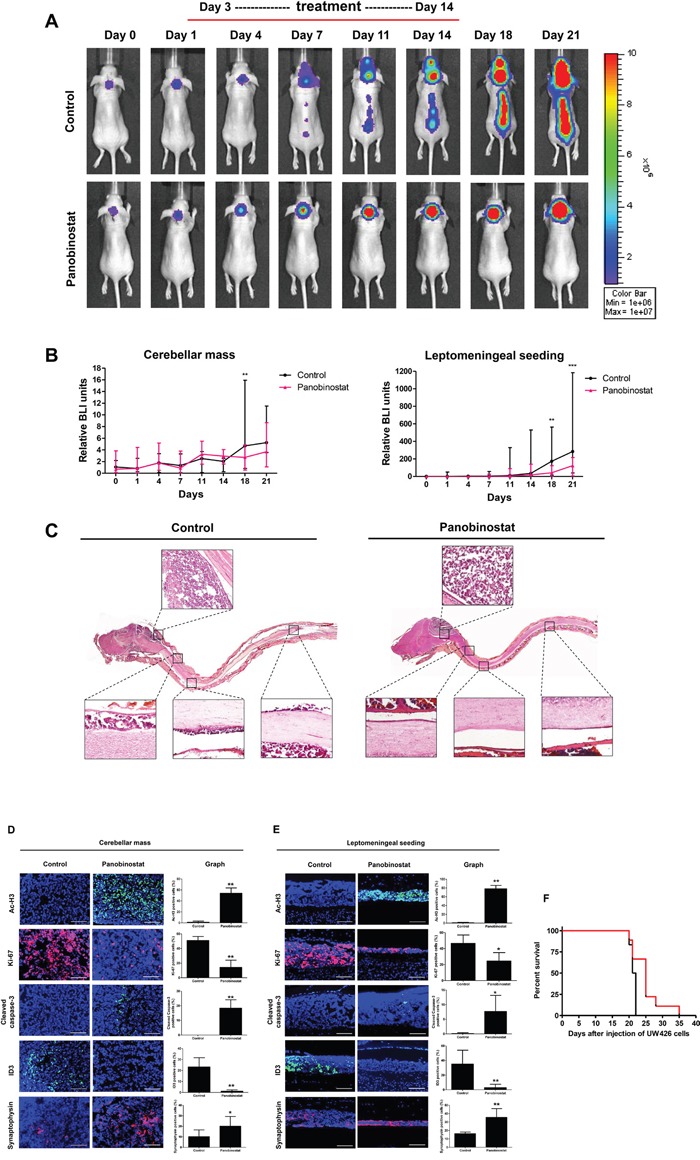
Therapeutic effect of panobinostat in a UW426-effLuc medulloblastoma leptomeningeal seeding model UW426-effLuc mice were treated 3 days later with panobinostat (10 mg/kg once daily for 2 weeks). **(A and B)** Serial bioluminescence image (BLI) of representative mice in each group. Quantification of BLI results shows that panobinostat reduced intracranial tumor burden and inhibited spinal cord seeding. **(C)** Representative longitudinal sections of whole craniospinal neuraxis of the mice with UW426-effLuc. Immunofluorescence with acetyl-H3 (green), Ki-67 (red), cleaved caspase-3 (green), ID3 (green) and synaptophysin (red). Representative images reveal increased Ac-H3, cleaved caspase-3 and synaptophysin and decreased Ki-67 and ID3 in **(D)** cerebellar mass and **(E)** leptomeningeal disseminated tumor cell layers. Scale bars, 100 μm. Cells were counterstained with 4′,6′-diamidino-2-phenylindole (blue). **(F)** A plot of overall survival is estimated by the Kaplan-Meier method. Panobinostat treatment significantly enhances the survival of mice bearing UW426-effLuc. *p<0.05, **p<0.01, ***p<0.001.

To evaluate the therapeutic effect of panobinostat, an MB leptomeningeal seeding animal model was established using UW426-effLuc cells as previously described [6]. We assessed the influence of panobinostat on MB leptomeningeal seeding in the whole craniospinal neuraxis. The stability of tumor cell engraftment, tumor growth and tumor cell seeding were monitored by bioluminescence imaging (BLI) with quantitative analysis (Figure 5A). Panobinostat modestly suppressed the growth of the primary tumor mass at the dorsal cerebellar surface only at the later stages of the experiment (p=0.012 at 18 days and p=0.104 at 21 days). However, the spinal seeding of the tumor was effectively suppressed in the panobinostat-treated group in all periods after spinal seeding appeared in the imaging (p=0.005 at 18 days and p<0.001 at 21 days; Figure 5B and Table 2). There was no mortality or obvious toxicity definitely attributable to the administration of panobinostat.

**Table 2:** Relative bioluminescence imaging (BLI) units

Groups		Day 14	Day 18	Day 21
Cerebellar mass	Control	1.79 ± 0.72	5.81 ± 4.98	5.60 ± 2.37
	Panobinostat	2.91 ± 0.66	2.60 ± 1.23*	4.34 ± 3.13
Leptomeningeal seeding	Control	134.85 ± 174.69	222.80 ± 187.80	479.67 ± 390.53
	Panobinostat	35.26 ± 43.15	46.57 ± 41.59**	107.50 ± 80.08***

*p<0.05, **p<0.01, ***p<0.001

A histological examination of the whole craniospinal neuraxis was performed. In control mice, we observed huge tumor masses at the dorsal surface of the cerebellum (at the injection site) and thin tumor cell layers diffusely situated on the leptomeninges from the subfrontal area to the lumbosacral spinal cord (Figure 5C). The tumor tissue showed a highly cellular, small round blue cell tumor composed of cells with large nuclei and scant cytoplasm, reminiscent of human MB. In the panobinostat-treated mice, the tumor mass at the dorsal cerebellum was also observed, but the diffuse layers of leptomeningeal seeding were significantly reduced.

In the panobinostat-treated group, the tumor cells exhibited a robust expression of Ac-H3 at the dorsal cerebellum (control vs. panobinostat: 1.00 ± 2.29 vs. 54.00 ± 9.71, p=0.004) and the leptomeningeal layer of the spinal cord (control vs. panobinostat: 0.89 ± 0.78 vs. 78.22 ± 7.65, p=0.009). A decreased number of Ki-67 positive cells and an increased number of cleaved caspase-3 cells were observed in the cerebellar mass (control vs. panobinostat: Ki-67, 51.01 ± 5.55 vs. 14.22 ± 9.92, p=0.008; cleaved caspase-3, 0.00 ± 0.00 vs. 18.33 ± 5.75, p=0.009; Figure 5D) and the leptomeningeal seeding (control vs. panobinostat: Ki-67, 46.56 ± 10.41 vs. 24.56 ± 10.43, p=0.013; cleaved caspase-3, 0.11 ± 0.33 vs. 7.67 ± 5.53, p=0.022; Figure 5E). The number of ID3 positive tumor cells was reduced in the cerebellar mass (control vs. panobinostat: ID3, 23.22 ± 8.45 vs. 1.11 ± 1.27, p=0.009; Figure 5D) and the leptomeningeal seeding (control vs. panobinostat: ID3, 35.11 ± 19.02 vs. 2.89 ± 4.68, p=0.009). In contrast, the number of synaptophysin positive cells was increased in both the cerebellar mass (control vs. panobinostat: 10.22 ± 6.38 vs. 20.22 ± 9.16, p=0.004; Figure 5D) and the leptomeningeal seeding (control vs. panobinostat: 16.23 ± 1.92 vs. 35.67 ± 10.30, p=0.008; Figure 5E).

There was a significant survival gain in the panobinostat-treated group (median survival, 25 days) over the control group (median survival, 21.5 days, p=0.039; Figure 5F).

## DISCUSSION

We demonstrated that the HDAC inhibitor panobinostat can exert suppressive effects on MB leptomeningeal seeding. *In vitro*, panobinostat inhibited the migration and adhesion capabilities of MB through the down-regulation of ID3 and the induction of neuronal differentiation. *In vivo*, panobinostat effectively suppressed the seeding of tumor cells in the spinal canal and significantly prolonged the survival of the mouse.

The clinical impact of MB leptomeningeal seeding is enormous. Approximately 1/3 of patients with MB present with leptomeningeal seeding at diagnosis [3]. Leptomeningeal seeding at presentation warrants reinforced chemotherapy and radiation therapy with higher toxicity, because it is related to higher rates of treatment failure, recurrence, and mortality. Moreover, recurrent MB is frequently accompanied by leptomeningeal seeding, which is robustly resistant to any therapies. The mortality of patients with recurrent MB reaches almost 90% [19]. Although the development of therapeutics for MB leptomeningeal seeding is strongly needed, the biology of this phenomenon is poorly understood.

It is argued that epigenetic adaptions of tumor cells allow for cellular plasticity and lead to cancer metastasis [20]. The genetic basis of MB has been well elucidated. Researchers have successfully made a variety of genetically engineered mouse (GEM) models in which MB-like tumors develop spontaneously at high penetrance [21]. The MB-GEM models depend on the disruption of canonical MB-related genetic pathways (sonic hedgehog or WNT) combined with knockout of key tumor suppressor genes, such as p53 and Kip1. Interestingly, only a few MB-GEM models develop spontaneous leptomeningeal seeding and with low fidelity at best [21]. This fact raises a suspicion that MB leptomeningeal seeding arises beyond the underlying genetic derangement, arguably by epigenetic alterations. Clinically, among the four molecular subtypes of MB, the Group 3 subtype is most frequently associated with leptomeningeal seeding. To date, no driver mutation has been found in Group 3 MB, and there is some evidence that Group 3 MB is an epigenetically driven tumor [22]. To overcome challenges in MB leptomeningeal seeing, the focus of studies needs to shift towards identifying epigenetic targets and novel therapeutics.

HDAC inhibitors may be a promising option for this situation. Previous studies supported the suppressive effects of HDAC inhibitors on brain tumor cell lines, including Group 3 MB cells [13, 23]. The expression of HDAC5 and HDAC9 was highly expressed in unfavorable subgroups of MB [24], suggesting HDAC inhibitors for novel candidate drugs.

Panobinostat is a powerful pan-HDAC inhibitor that received U.S. Food and Drug Administration (FDA) and European Medical Agency (EMA) approval for multiple myeloma treatment in 2015 [9]. Panobinostat regulates hallmarks of cancer cell biology, such as the cell cycle progression, apoptosis and angiogenesis [25, 26]. Our results not only show the regulation of target proteins relevant to MB leptomeningeal seeding but also demonstrate the feasibility of the clinically relevant usage of panobinostat through *in vivo* experiments. However, although the suppressive actions of panobinostat against MB leptomeningeal seeding were very powerful both *in vitro* and *in vivo*, the survival gain was modest. From these findings, it can be suggested that panobinostat is largely selective in its anti-cancer effect, suppressing MB leptomeningeal seeding without eliminating bulk tumors. This may be a common phenomenon of epigenetic modulator drugs with which only a small portion of patients experiences complete remission of target disease [27, 28]. In our study, we observed robust neuronal differentiation after panobinostat treatment. Theoretically, differentiation therapy is a strategy to reprogram, adapt, and tame haphazard cancer cells into the control of a normal regulatory network, without eliminating them immediately. A combination of conventional anti-cancer agents with panobinostat would be a more effective treatment because these drugs may act synergistically to reverse various cancer hallmark characteristics [8].

## MATERIALS AND METHODS

### Cell cultures and HDAC inhibitor

Established MB cell lines (UW228, UW426, and MED8A) were provided by Young-Shin Ra at the Asan Medical Center, Seoul, Korea. The established UW228 and UW426 cell lines have been classified as sonic hedgehog, MED8A as Group 3 [29]. The cells were cultured in DMEM supplemented with 10% FBS and 1% penicillin/streptomycin at 37°C in a humidified incubator set at 5% CO_2_. Panobinostat (LBH589) was purchased from Selleckchem (Houston, TX) and dissolved in dimethyl sulfoxide (DMSO; Sigma Aldrich, St. Louis, MO).

### Cell viability and proliferation

The cells (4×10^3^ cells/well) were seeded in a 96-well plate and treated with different concentrations of panobinostat for 24, 48 and 72 h. The cell viability was detected using Cell Counting Kit-8 (CCK; Dojindo, Kumamoto, Japan) and was measured at 450 nm using a micro-ELISA reader (Molecular Devices, Sunnyvale, CA). The percentage of cellular viability was averaged and normalized against the untreated control (DMSO). The half maximal inhibitory concentration (IC_50_) values were estimated for further studies. The cells were seeded (1 × 10^4^/well) on 8-well chamber slides (Nunc, Rochester, NY) and fixed. For the proliferation analysis, the fixed cells were incubated with 5-ethynyl-2′-deoxyuridine (EdU) using the Click-it EdU assay kit (Invitrogen, Carlsbad, CA) at 48 h, according to the manufacturer's protocol. The results were analyzed by a confocal microscope (Carl Zeiss, Jena, Germany). EdU-positive cell number against the total cell number.

### Cell cycle progression

The cells were fixed in 70% iced-cold ethanol and were incubated for 1 h at 4°C. The cells were washed, resuspended with 0.5 mg/ml RNase A (Sigma-Aldrich) for 1 h at 37°C and a 10 μg/ml propidium iodine solution (Sigma-Aldrich) was added in the dark at 4°C. The cells were observed by fluorescence microscopy and were analyzed using fluorescence-activated cell sorting (FACS).

### Apoptosis analysis by immunofluorescence

The cells (1 × 10^4^/well) were seeded on 8-well chamber slides (Nunc), treated with panobinostat, fixed, permeated and blocked as described previously [6]. Anti-cleaved caspase-3 (1:100; Abcam, Cambridge, MA) was used as the primary antibody and the Alexa Fluor 488-conjugated goat anti-rabbit IgG (1:500; Invitrogen) was the secondary antibody. The slides were mounted with an antifading solution containing 4′-6′-diamidino-2-phenyl-indole (Vector Laboratories, Burlingame, CA). Images were taken using a confocal microscope (Carl Zeiss). Cleaved caspase-3-positive cell number against the total cell number.

### Immunoblotting

Total protein was extracted and a western blot was performed as described previously [30]. The following antibodies were used: anti-Ac-H3 (1:1,000; Abcam), anti-cleaved caspase-3 (1:200; Millipore, Billerica, MA), anti-CCND1 (1:1,000; Abcam), anti-ID1 (1:500; Cell Signaling Technology, Danvers, MA), anti-ID2 (1:500; Cell Signaling Technology), anti-ID3 (1:500; Cell Signaling Technology), anti-ID4 (1:250; Cell Signaling Technology), anti-synaptophysin (1:1,000; Abcam), anti-NeuroD1 (1:1,000; Abcam) and anti-β-actin (1:10,000; Sigma- Aldrich).

### Cell migration and adhesion assay

Cell migration and adhesion were assessed using the CytoSelect 48-well cell migration and adhesion assay kit (Cell Biolabs, San Diego, CA) according to the manufacturer's protocol.

### Differentiation analysis by immunofluorescence

Anti-synaptophysin (1:500; Abcam) and anti-NCAM (1:500; Abcam) were used as the primary antibodies and the Alexa Fluor 594-conjugated goat anti-rabbit IgG (1:500; Invitrogen) was the secondary antibody. The slides were mounted with an antifading solution containing 4′-6′-diamidino-2-phenyl-indole (Vector Laboratories, Burlingame, CA). Images were taken using a confocal microscope (Carl Zeiss, Jena, Germany).

### Retroviral infection

For *in vivo* live imaging, the UW426 cells were infected by the enhanced firefly luciferase gene (effLuc) as described previously [31]. After magnetic-activated cell sorting (MACS; Miltenyi Biotech, Bergisch Gladbach, Germany) of the UW426-effLuc, the luciferase activity was confirmed using a luciferase assay kit (Promega, Madison, WI) according to the manufacturer's protocol.

### Mouse model of MB leptomeningeal seeding

Female BALB/c nude mice (7 weeks old) were purchased from Orient Bio Inc. (Sungnam, Korea). The Institutional Animal Care and Use Committee of Seoul National University Hospital (SNUH) approved all of the animal experiment protocols [SNUH-IACUC No.14-0206-C1A0]. The mice were anesthetized by an intraperitoneal (i.p.) injection of 20 mg/kg Zoletil (Virbac, Carros, France) and 10 mg/kg Rompun (Bayer, Leverkusen, Germany). After the cisterna magna was exposed, the UW426-effLuc (1.2 × 10^6^ cells) cells were slowly injected into the subarachnoid space of the cisterna magna using a 30-gauge needle as previously described [32].

### *In vivo* experimental design and bioluminescence imaging (BLI)

To determine the effect of panobinostat *in vivo*, a mouse model of MB leptomeningeal seeding was used. Three days after the injection of the UW426-effLuc cells into the cisterna magna, the mice were randomly divided into 2 groups (N=8 per group). The control group received DMSO in 0.5% dextrose and the panobinostat group received 10 mg/kg of panobinostat every 5 days for 2 weeks. For the *in vivo* BLI analysis, the mice were sedated with 2% isoflurane in 100% O_2_ through a nose cone. After D-Luciferin (150 mg/kg, Caliper Life Sciences) was administered, bioluminescence images were taken by an *In-Vivo* Imaging System 100 (Xenogen Corp.) on days 0, 1, 4, 7, 11, 14, 18 and 21, and the images were analyzed as described previously [31, 32]. For the survival analysis, the mice were followed until they died (control vs. treatment, N=8 per group). The development of severe symptoms and signs requiring euthanasia were considered as mortality, and all of the mice were verified as bearing tumors by necropsy.

### Histopathological and immunofluorescence analysis

For the histopathological analysis, the mice were sacrificed 18 days after the UW426-effLuc injection (control vs. treatment, N=7 per group). After perfusion under deep anesthesia, the whole brain was harvested and prepared for frozen sectioning as previously reported [32]. The sections were stained with hematoxylin and eosin. For the immunofluorescence analysis, anti-Ac-H3 (1:100; Abcam), anti-Ki-67 (1:200; Abcam), anti-cleaved caspase-3 (1:100; Abcam), anti-ID3 (1:100; Cell Signaling Technology) and anti-Synaptophysin (1:100; Abcam) were used with previously described protocols [32]. The images were acquired by a Leica fluorescence microscope (Leica Microsystems, Bannockburn, IL). The number of positive cells within 100 nuclei was counted from 3 randomly selected high-power fields in each section using Leica application suite software.

### Statistical analysis

All experiments and analyses were performed in triplicate. The values are presented as the mean ± standard deviation (SD). A Mann–Whitney U test or Student's t-test was applied to compare the continuous variables between two groups. The survival in each group was analyzed using the Kaplan-Meier method. A log-rank test was used for comparisons of the survival data. P <0.05 was considered statistically significant. The statistical analyses were conducted using IBM-SPSS version 21.0 software (IBM-SPSS, Armonk, NY).
